# Fairness Views, Equal Sharing, and Contributions in Team Production

**DOI:** 10.3390/bs16101790

**Published:** 2026-10-01

**Authors:** Yanan Zhang, Yaxin Zhang, Xiaoyu Zhang

**Affiliations:** 1 Business School/Institute of New Structure Industry Development, Shandong Normal University, Jinan 250358, China; zhangyn@sdnu.edu.cn; 2 School of Economics, Qufu Normal University, Rizhao 276826, China; yxzhang@qfnu.edu.cn; 3 School of Health Management, Shandong University of Traditional Chinese Medicine, Jinan 250355, China

**Keywords:** fairness views, public goods game, team production, equal sharing

## Abstract

Previous research has documented heterogeneity in individuals’ fairness views regarding how jointly produced output should be distributed. Using a public goods game as a simplified representation of collective production, we report three experiments examining whether individuals’ fairness views are associated with their contributions under an equal-sharing rule (Experiment 1), whether fairness-related prompting can affect contributions (Experiment 2), and whether the procedure used to determine participation under the arrangement affects rule acceptance and contributions (Experiment 3). Experiment 1 shows that participants with more egalitarian fairness views contributed more under the equal-sharing rule, while Experiment 2 provides causal evidence that fairness-related prompting affected subsequent contributions: participants asked to generate reasons why equal sharing is fair contributed more than those asked to generate reasons why it is unfair. Experiment 3 shows that, compared with a procedure in which participation was imposed, a procedure in which participation was determined by group voting resulted in higher contributions and greater acceptance of the equal-sharing rule. Exploratory analyses further suggest that the effects of voting may be stronger among participants with less egalitarian initial fairness views. Our findings indicate that individuals’ fairness views and the procedures used to determine participation are relevant to understanding contribution behavior under an equal-sharing rule.

## 1. Introduction

Team production is a common form of work organization (Mathieu et al., 2008). An important issue in team production is how jointly produced output or team rewards should be allocated among team members (Nalbantian & Schotter, 1997; Lazear, 2018; Angelovski et al., 2021; Freeman et al., 2025). Economic theory typically evaluates the effectiveness of different allocation rules in motivating individual effort from the perspective of their incentive properties: different allocation rules create different links between individual input and individual reward and, consequently, different incentives for team members to exert effort (Holmström, 1982; Lazear, 2018). For example, under an equal-sharing rule, individual earnings are only weakly tied to individual contributions. This weak link may encourage free riding and thereby reduce team efficiency. By contrast, contribution-based allocation strengthens the link between individual effort and reward and can therefore provide stronger incentives for team members to contribute to production (Balafoutas et al., 2013; Boosey et al., 2024).

However, the effectiveness of an allocation rule may depend not only on its objective incentive properties but also on how it is evaluated by the individuals subject to it. Previous research shows substantial heterogeneity in individuals’ fairness ideals—that is, their views of what constitutes a fair distribution (Cappelen et al., 2007, 2010; Luhan et al., 2019; Almås et al., 2020, 2025a, 2025b, 2025c; Müller & Renes, 2021). Such heterogeneity may have behavioral consequences for contribution decisions in collective production settings. The literature on moral preferences suggests that individuals are partly motivated to choose actions they perceive as morally appropriate, even when doing so does not maximize their material payoff (Tappin & Capraro, 2018; Bašić & Verrina, 2024). If fairness ideals shape how individuals evaluate a particular allocation rule, they may also shape what level of contribution individuals regard as appropriate under that rule. Thus, individuals holding different fairness views may respond differently to the same allocation arrangement, even when they face identical material incentives.

Against this background, this paper examines whether individuals’ fairness views are related to their contributions under an equal-sharing rule. We further examine whether fairness-related prompting changes subsequent contributions and whether the procedure through which participation under the arrangement is determined affects rule acceptance and contributions. Beyond compensation design within firms, these questions also speak to broader themes in sustainable development, a multidimensional concept encompassing economic, social, and environmental dimensions (Klarin, 2018; Manioudis & Meramveliotakis, 2022). In particular, our study relates to these economic and social dimensions by examining how people contribute to collective production, respond to equal-sharing arrangements, and participate in collective decision-making. These themes relate to the United Nations Sustainable Development Goals concerning sustained, inclusive and sustainable economic growth (SDG 8), reduced inequalities (SDG 10), and inclusive institutions and participatory decision-making (SDG 16) (United Nations, 2015).

This study addresses these questions using a public goods game, in which returns from the public account are shared equally among group members. This setting provides a simple representation of team production under an equal-sharing rule. Experiment 1 measures participants’ fairness views regarding equal sharing and examines how these views are associated with their contributions in the public goods game. We find that participants who hold more egalitarian fairness ideals contribute more to the public account. Given the possibility suggested by Experiment 1 that fairness-related evaluations of the rule may matter for contribution, Experiment 2 further examines whether fairness-related prompting concerning the equal-sharing rule can affect contributions. Participants are randomly assigned to generate reasons why equal sharing is fair or unfair before making their contribution decisions. Those prompted to consider equal sharing fair subsequently contribute more than those prompted to consider it unfair. Experiment 2 therefore provides causal evidence that fairness-related prompting can affect contribution behavior.

Experiment 3 extends the analysis by turning from individuals’ evaluations of the equal-sharing rule to the procedure through which participation under the arrangement is determined.^1^ Research on procedural justice suggests that individuals’ acceptance of an arrangement may depend not only on its distributive content but also on the procedure through which it is established (Thibaut & Walker, 1975; Lind & Tyler, 1988; Tyler, 2006). In particular, participatory and democratic decision procedures may enhance the perceived legitimacy and acceptance of an allocation arrangement even when its distributive content remains unchanged. We therefore compare a setting in which participation under the equal-sharing rule is imposed exogenously with one in which participation is determined through a democratic voting procedure, while holding the allocation rule itself fixed. We find that participants in the voting condition contribute more and report greater acceptance of the equal-sharing rule. Exploratory analyses further suggest that these effects are larger among participants with less egalitarian pre-existing fairness views.

This study makes three contributions to the literature. First, our study complements the traditional economic emphasis on the incentive properties of allocation schemes by linking fairness views to contributions under equal sharing. Economic analyses typically emphasize the objective incentive properties of allocation schemes, such as the link between individual effort and reward and the incentives for free riding (Holmström, 1982; Lazear, 2018). Our findings show that contribution behavior under an equal-sharing rule is also related to individuals’ fairness views. Related evidence is provided by Capraro et al. (2014), whose findings suggest that egalitarian cooperative choices can be associated with fairness considerations. Unlike our study, however, they did not separately measure participants’ fairness views, but instead inferred fairness considerations from participants’ responses to a post-experiment open-ended question.

Second, our study contributes to the literature on compensation schemes by highlighting heterogeneity in individuals’ responses to the same scheme. Much of the existing literature compares the average effectiveness of different compensation schemes (e.g., Nalbantian & Schotter, 1997; Lazear, 2000; van Dijk et al., 2001; Hamilton et al., 2003; Dohmen & Falk, 2011; Angelovski et al., 2021; Freeman et al., 2025). In contrast, we hold the allocation rule fixed and examine whether responses under the same equal-sharing rule vary with fairness views and rule acceptance. We show that participants with more egalitarian fairness views and greater rule acceptance contribute more under equal sharing. This perspective suggests that not only do different compensation schemes vary in their effectiveness, but the same scheme may also have different effects across individuals.

Third, our study contributes to the literature on fairness ideals by linking how a distributive arrangement is determined to individuals’ evaluations of the arrangement. A substantial literature on fairness ideals has examined which distributions individuals regard as fair and documented considerable heterogeneity in these judgments (Cappelen et al., 2007, 2010; Rodriguez-Lara & Moreno-Garrido, 2012; Ubeda, 2014; Luhan et al., 2019; Almås et al., 2020, 2025a, 2025b, 2025c; Müller & Renes, 2021). We show that evaluations of a given allocation arrangement may also depend on the procedure used to determine participation under it. In our experiment, allowing participants to determine participation under the equal-sharing rule through a democratic voting procedure increases both the perceived fairness of the rule and contributions under it. These findings suggest that fairness judgments may be shaped not only by the substantive features of an allocation rule but also by the procedure used to determine participation under it.

The remainder of the paper is organized as follows. Section 2, Section 3 and Section 4 present the designs and results of the three experiments. Section 5 discusses the main findings, practical implications, and limitations of this study, and Section 6 concludes.

## 2. Experiment 1

### 2.1. Experimental Design

#### 2.1.1. Public Goods Game

We used a public goods game to represent team production under an equal-sharing rule. Participants played a one-shot, four-person linear public goods game. Each participant received an endowment of 20 experimental tokens and chose an integer contribution *g_i_* between 0 and 20 to a public account. Total contributions were doubled and divided equally among the four group members. Participant *i*’s payoff was therefore calculated as follows:

*π_i_*(*g*_1_, …, *g*_4_) = 20 − *g_i_* + 0.5 *∑_j_*_=1_^4^
*g_j_*. (1)

Thus, contributing increased total group earnings but reduced the contributor’s private payoff when holding others’ decisions constant. The contribution decision serves as our measure of individual contribution to team production.

#### 2.1.2. Fairness-Ideal Elicitation

We used two items from the fairness-ideal questionnaire developed by Luhan et al. (2019) to measure the extent to which participants held an egalitarian fairness ideal. The original questionnaire was designed to distinguish among four fairness ideals: libertarianism, meritocracy, choice egalitarianism, and egalitarianism. It contained seven items, including four open-choice questions and three multiple-choice questions. Because the allocation rule examined in our public goods game divides jointly produced output equally, our analysis focuses specifically on the extent to which participants’ fairness judgments are consistent with the egalitarian principle. Specifically, we selected one open-response item and one multiple-choice item from their questionnaire to construct our measure of consistency with the egalitarian ideal.^2^ In both items, participants were asked to allocate a joint output between two hypothetical individuals, A and B, without having any personal stake in the allocation. Neither question was incentivized. Each hypothetical individual’s output was decomposed into three sources—talent, effort, and luck—allowing participants’ allocation judgments to reflect their views about which sources of production should legitimately determine individual earnings.

In Question 1, participants were shown the contributions of A and B from talent, effort, and luck and were asked to specify what they considered a fair allocation of the joint output. No candidate allocations were provided, so participants could state their preferred allocation without being influenced by predefined alternatives. The two amounts entered by participants were required to sum to the total output of 33. The numerical values used in this question are reported in Table 1.

In Question 2, participants were again shown the contributions of A and B from talent, effort, and luck, but were asked to select the fairest allocation from four predefined alternatives. Following the classification of fairness ideals in Cappelen et al. (2010), Options 1–4 corresponded to libertarian, meritocratic, choice-egalitarian, and egalitarian allocation principles, respectively. The production components and allocation options are reported in Table 2 and Table 3.

Following Luhan et al. (2019), we constructed an egalitarian index for each participant *i* using their responses to the two questions. For Question 1, the egalitarian score was *F_i_*_1_ = 1 − |*m_a_* − *f_a_*|/*f_a_*, where *m_a_* is the amount assigned to A and *f_a_* = 16.5 denotes the amount A would receive under equal sharing. For Question 2, *F_i_*_2_ equaled 1 when the equal allocation (Option 4) was selected and 0 otherwise. The overall egalitarian index was *F_i_* = (*F_i_*_1_ + *F_i_*_2_)/2. A higher value of *F_i_* therefore indicates greater consistency between a participant’s fairness ideal and the egalitarian principle underlying equal sharing.

#### 2.1.3. Procedure

The experiment proceeded in several stages. Participants first completed the two fairness-ideal elicitation questions described in Section 2.1.2. These decisions were made before participants were introduced to the public goods game and were intended to measure their general views about fair allocation independently of the specific allocation rule used in the subsequent game.

Participants then read the instructions for the public goods game. After reading these instructions but before making their contribution decisions, participants rated the fairness of the equal-sharing rule used in the game by answering the question “To what extent do you think it is fair to divide the funds in the public account equally among all group members?” Responses were recorded on a seven-point scale ranging from 1 (very unfair) to 7 (very fair). We label this variable rule acceptance. Unlike the egalitarian index, which captures participants’ general fairness ideals, rule acceptance measures their fairness evaluation of the specific equal-sharing rule implemented in the public goods game. We therefore use rule acceptance as a context-specific measure of participants’ evaluation of the equal-sharing rule and report it as a supplementary robustness check alongside analyses based on the egalitarian index.

Participants next made their incentivized contribution decision by choosing how many of their 20 tokens to contribute to the public account. After making this decision, they reported how many tokens they expected the other three group members to contribute on average. We elicited this expectation because contribution decisions in public goods games may depend on beliefs about others’ contributions (Ackermann & Murphy, 2019).^3^

Participants then answered two comprehension questions designed to assess their understanding of the rules of the public goods game. Participants who failed the comprehension test were excluded from the subsequent analysis. After completing the experimental tasks, participants reported their general willingness to take risks on a 1-to-10 scale adapted from Dohmen et al. (2011) and provided demographic information, including gender, age, family residence (urban or rural), and household per capita monthly income. Household income was recorded in five categories: Chinese yuan (CNY) 2000 or less, CNY 2001–4000, CNY 4001–6000, CNY 6001–8000, and above CNY 8000. Risk preference and these demographic characteristics were included as control variables in the subsequent regression analyses.

The experiment was conducted online using Wenjuanxing (web-based platform; Changsha Ranxing Information Technology Co., Ltd., Changsha, China), a widely used online survey platform in China. The complete experimental instructions for all three experiments, translated from Chinese, are provided in Appendix A. Participants were recruited from the student population of Shandong University. A total of 160 participants completed the study. After excluding 37 participants who failed the comprehension test, the final sample consisted of 123 participants. The final sample included 62 women (50.407%) and 61 men (49.593%), with a mean age of 21.553 years (*SD* = 3.006). 76 participants (61.789%) reported an urban family location, and the median category of household per capita monthly income was CNY 4001–6000.^4^ To prevent repeated participation in Experiment 1, the Wenjuanxing link was configured to allow for only one submission per device and per account. We also checked the Alipay account identifiers provided for payment and found no duplicates in the Experiment 1 sample.

Because Wenjuanxing did not support real-time participant matching, group formation and earnings calculations were conducted after data collection, using only participants who had passed the comprehension test. Because the number of eligible participants was not necessarily divisible by four, we did not partition them into mutually exclusive four-person groups. Instead, we constructed a separate group for each eligible participant solely for the purpose of calculating that participant’s earnings. Specifically, for each focal participant, three distinct participants were randomly selected from the remaining pool of eligible participants. The decisions of the focal participant and the three selected participants were then used to calculate the focal participant’s earnings. This procedure was repeated separately for every eligible participant. Experimental tokens were converted into cash at a rate of 10 tokens to CNY 1. In addition to their experimental earnings, each participant received a fixed participation fee of CNY 1. The total payment was transferred to the Alipay account provided by the participant.

### 2.2. Results

Table 4 presents regression estimates of the association between participants’ egalitarian fairness views and their contributions in Experiment 1. Columns (1) and (2) report ordinary least squares (OLS) estimates. When no additional control variables were included, the egalitarian index was positively and statistically significantly associated with contribution (*b* = 11.686, *SE* = 1.794, standardized *β* = 0.375, *p* < 0.001, 95% CI [8.133, 15.239]). The association remained positive and statistically significant after controlling for individual characteristics (*b* = 12.331, *SE* = 2.022, standardized *β* = 0.395, *p* < 0.001, 95% CI [8.326, 16.336]). Because contributions were bounded between 0 and 20, we also estimated two-limit Tobit models, as reported in columns (3) and (4). The Tobit estimates were consistent with the OLS results. The coefficient of the egalitarian index was positive and statistically significant both in the specification without additional controls (*b* = 17.916, *SE* = 3.910, *p* < 0.001, 95% CI [10.252, 25.580]) and in the specification with controls for individual characteristics (*b* = 19.012, *SE* = 4.146, *p* < 0.001, 95% CI [10.886, 27.138]).

Appendix B.1 reports a series of supplementary analyses for Experiment 1. These analyses include participants who failed the comprehension check, control for expected peer contribution, use rule acceptance as an alternative measure of participants’ evaluation of the equal-sharing rule, examine the two components of the abbreviated index separately, and replace the abbreviated index with the full seven-item index. The positive association between egalitarian fairness views and contribution remained substantively unchanged. Taken together, the results provide robust evidence that participants with stronger egalitarian fairness views contributed more under the equal-sharing rule used in Experiment 1.

## 3. Experiment 2

Experiment 1 documented a positive association between participants’ egalitarian fairness views and their contributions under the equal-sharing rule, suggesting that fairness-related evaluations of the rule may be related to contribution behavior. Building on this possibility, Experiment 2 further examines whether fairness-related prompting concerning equal sharing affects subsequent contributions.

### 3.1. Experimental Design

Participants were randomly assigned to either the fair-prompt or the unfair-prompt condition.^5^ In the fair-prompt condition, participants were asked to consider and write down reasons why equal sharing is fair. In the unfair-prompt condition, participants were asked to consider and write down reasons why equal sharing is unfair.

Following the writing task, participants completed the same two fairness-ideal elicitation questions used in Experiment 1. They then read the instructions for the public goods game and rated the fairness of the equal-sharing rule on the same seven-point scale. The egalitarian index and rule-acceptance measure were used to assess whether the two prompting conditions differed in subsequent fairness-related evaluations. Participants subsequently played the same one-shot, four-person public goods game as in Experiment 1. After making their contribution decisions, they reported their expected peer contribution, completed the two comprehension questions, and provided the same risk preference and demographic measures as in Experiment 1.

The experiment was conducted online using the Wenjuanxing platform, with participants recruited from Shandong University. A total of 332 participants completed the experiment: 165 in the fair-prompt condition and 167 in the unfair-prompt condition. Of these participants, 82 failed the comprehension test and were excluded, including 47 in the fair-prompt condition and 35 in the unfair-prompt condition. The final analytic sample therefore consisted of 250 participants, including 118 in the fair-prompt condition and 132 in the unfair-prompt condition. Participants had a mean age of 21.200 years (*SD* = 2.680); 93 participants (37.200%) were male, and 131 (52.400%) came from urban households. The median category of household per capita monthly income was CNY 4001–6000. Students who had participated in Experiment 1 were ineligible for Experiment 2, and each participant could complete Experiment 2 only once.^6^ Earnings were calculated after the experiment using the same random-matching and payment procedures as in Experiment 1, with matching restricted to participants in the same experimental condition.

### 3.2. Results

#### 3.2.1. Randomization Check

To examine comparability between the two experimental conditions, we conducted balance checks using Mann–Whitney U tests for continuous and ordinal variables and Pearson’s chi-square tests for categorical variables. No statistically significant differences were found in age (*p* = 0.492), family location (*p* = 0.833), household income category (*p* = 0.625), or risk preference (*p* = 0.247). However, the proportion of male participants was lower in the fair-prompt condition than in the unfair-prompt condition (31.356% versus 42.424%), with the difference significant at the 10% level (*p* = 0.071). To assess the robustness of the unadjusted estimates to observed covariate differences, we also report adjusted specifications controlling for gender, age, family location, household income category, and risk preference.

#### 3.2.2. Manipulation Check

We next examined whether the two prompting conditions differed in fairness-related evaluations. The mean egalitarian index was 0.511 (*SE* = 0.019) in the fair-prompt condition and 0.440 (*SE* = 0.011) in the unfair-prompt condition. The difference was statistically significant according to the Mann–Whitney U test (*p* = 0.019). Mean rule acceptance was also higher in the fair-prompt condition (*M* = 3.839, *SE* = 0.146) than in the unfair-prompt condition (*M* = 3.432, *SE* = 0.113), and this difference was marginally significant at the 10% level (*p* = 0.061). Overall, the manipulation checks support the intended difference between the two prompting conditions.

#### 3.2.3. Effects of Fairness Prompting on Contributions

The mean contribution was 11.873 tokens (*SE* = 0.613) in the fair-prompt condition and 9.652 tokens (*SE* = 0.493) in the unfair-prompt condition. The Mann–Whitney U test indicated that this difference was statistically significant (*p* = 0.006; Cohen’s *d* = 0.361). Table 5 presents the regression estimates. Column (1) reports the primary OLS estimate without additional control variables. Participants in the fair-prompt condition contributed, on average, 2.221 more tokens than those in the unfair-prompt condition (*b* = 2.221, *SE* = 0.787, standardized *β* = 0.178, *p* = 0.005, 95% CI [0.672, 3.771]). Column (2) additionally controls for risk preference, gender, age, family location, and household income category. The estimated difference between the two prompting conditions remained positive and statistically significant (*b* = 1.829, *SE* = 0.777, standardized *β* = 0.147, *p* = 0.019, 95% CI [0.299, 3.359]). Because contributions were bounded between 0 and 20, columns (3) and (4) report two-limit Tobit estimates. The coefficient on the fair-prompt condition indicator was positive and statistically significant both without additional controls (*b* = 3.522, *SE* = 1.184, *p* = 0.003, 95% CI [1.202, 5.842]) and with controls for individual characteristics (*b* = 2.960, *SE* = 1.145, *p* = 0.010, 95% CI [0.717, 5.203]).

Appendix B.2 reports two supplementary robustness analyses. First, the estimated treatment coefficient remained positive and statistically significant in both the OLS and Tobit models when participants who failed the comprehension check were included. Second, after additionally controlling for expected peer contribution, the estimated difference between the two prompting conditions remained positive and was marginally significant at the 10% level in the OLS model and statistically significant in the Tobit model.

Overall, Experiment 2 provides causal evidence that fairness-related prompting affects subsequent contribution behavior. Participants prompted to generate reasons why equal sharing is fair evaluated the rule more favorably and contributed more than those prompted to generate reasons why it is unfair.

## 4. Experiment 3

Experiment 3 shifts attention to the procedural dimension of the equal-sharing arrangement. We examine whether allowing participants to vote on participation, rather than having participation imposed exogenously, affects rule acceptance and contributions.

### 4.1. Experimental Design

Participants first completed the same two fairness-ideal elicitation questions used in Experiments 1 and 2. They then proceeded to the public goods game stage. The experiment used a between-participants design in which participants were randomly assigned to either the no-vote condition or the vote condition before receiving condition-specific instructions. The assignment procedure was the same as in Experiment 2. The two conditions differed in how the decision about participation in the public goods game under the equal-sharing arrangement was made. In the no-vote condition, all participants were required to participate in the same public goods game used in Experiments 1 and 2, so participation under the equal-sharing arrangement was imposed exogenously. In the vote condition, participants voted on whether their four-person group would participate in this game. The group decision was determined by majority rule, with ties resolved randomly. If the group voted to participate, participants’ earnings were determined by their decisions in the public goods game; otherwise, each group member received a fixed payoff of 20 tokens. Thus, in the vote condition, participation under the equal-sharing arrangement was determined democratically rather than imposed exogenously. We therefore regard the voting procedure as a more procedurally fair way of determining whether the equal-sharing arrangement would be implemented for the group.

After participants in the vote condition cast their votes, they answered the subsequent questions under the assumption that their group had voted to participate, regardless of the actual voting outcome. They rated the fairness of the equal-sharing rule in the public goods game, chose how many tokens to contribute to the public account, reported their expected peer contribution, and completed the same comprehension questions, risk preference measure, and demographic measures as in Experiments 1 and 2. By eliciting these decisions from all participants in the vote condition regardless of the realized voting outcome, we avoided restricting the sample to participants whose groups voted to participate. Because willingness to participate may be correlated with willingness to contribute, conditioning on realized participation could introduce systematic differences between the vote and no-vote samples and thereby confound the comparison of contributions across conditions. Participants in the no-vote condition completed the same sequence of tasks, except that they did not participate in the voting stage.

The experiment was conducted online using the Wenjuanxing platform, with participants recruited from Shandong University. A total of 208 participants completed the experiment: 118 in the vote condition and 90 in the no-vote condition. Of these participants, 68 failed the comprehension test and were excluded, including 36 in the vote condition and 32 in the no-vote condition. The final analytic sample therefore consisted of 140 participants: 82 in the vote condition and 58 in the no-vote condition. Participants’ mean age was 21.014 years (*SD* = 2.553). The sample included 59 male participants (42.143%), and 82 participants (58.571%) reported an urban family location. The median category of household per capita monthly income was CNY 4001–6000. Earnings were calculated after the experiment using the same random-matching and payment procedures as in Experiments 1 and 2, with matching restricted to participants in the same experimental condition. In the vote condition, participants received the payoff from the public goods game if the vote outcome of the randomly matched four-person group was to participate; otherwise, they received a fixed payoff of 20 tokens.

### 4.2. Results

#### 4.2.1. Randomization Check

To examine comparability between the vote and no-vote conditions, we conducted balance checks using Mann–Whitney U tests for continuous and ordinal variables and Pearson’s chi-square tests for categorical variables. No statistically significant differences were found in age (*p* = 0.452), gender (*p* = 0.396), family location (*p* = 0.992), household income category (*p* = 0.357), or risk preference (*p* = 0.244). We additionally included these individual characteristics as controls in supplementary regression analyses.

#### 4.2.2. Effects of Voting on Contributions

The results show that mean contribution was 13.061 tokens (*SE* = 0.716) in the vote condition and 10.345 tokens (*SE* = 0.774) in the no-vote condition. The Mann–Whitney U test indicated that this difference was statistically significant (*p* = 0.010; Cohen’s *d* = 0.435). Table 6 presents the corresponding regression estimates. Column (1) reports a positive and statistically significant coefficient of the vote-condition indicator in the OLS specification without additional control variables (*b* = 2.716, *SE* = 1.054, standardized *β* = 0.211, *p* = 0.011, 95% CI [0.632, 4.800]). Column (2) additionally controls for risk preference, gender, age, family location, and household income category. The estimated treatment effect remained positive and was marginally significant at the 10% level (*b* = 1.928, *SE* = 1.070, standardized *β* = 0.150, *p* = 0.074, 95% CI [−0.188, 4.044]). Columns (3) and (4) report Tobit specifications with lower and upper limits of 0 and 20. The estimated effect of voting was positive and statistically significant both without additional controls (*b* = 4.441, *SE* = 1.632, *p* = 0.007, 95% CI [1.242, 7.641]) and with controls for individual characteristics (*b* = 3.255, *SE* = 1.572, *p* = 0.038, 95% CI [0.175, 6.336]). Taken together, these results indicate that participants in the vote condition contributed more than those in the no-vote condition.

We next examined whether the voting procedure also affected rule acceptance. Mean rule acceptance was higher in the vote condition (*M* = 4.146, *SE* = 0.192) than in the no-vote condition (*M* = 3.534, *SE* = 0.212), with the difference marginally significant according to a Mann–Whitney U test (*p* = 0.067; Cohen’s *d* = 0.363). Consistent with this result, Table 7 shows that the coefficient of the vote-condition indicator was positive and statistically significant in the unadjusted OLS specification in column (1) (*b* = 0.612, *SE* = 0.286, standardized *β* = 0.177, *p* = 0.034, 95% CI [0.047, 1.177]) and remained positive and marginally significant at the 10% level after controlling for individual characteristics in column (2) (*b* = 0.518, *SE* = 0.282, standardized *β* = 0.150, *p* = 0.069, 95% CI [−0.040, 1.077]). Columns (3) and (4) report ordered logit specifications to account for the ordinal nature of the seven-point rule-acceptance measure. The coefficient of the vote-condition indicator was positive and marginally significant both without additional controls (*b* = 0.559, *SE* = 0.303, *p* = 0.065, 95% CI [−0.035, 1.152]) and with individual-characteristic controls (*b* = 0.539, *SE* = 0.305, *p* = 0.077, 95% CI [−0.058, 1.137]). Thus, participants in the vote condition both contributed more and reported greater acceptance of the equal-sharing rule than participants in the no-vote condition.

We next examined whether the effects of voting varied with participants’ pre-treatment fairness views. If voting helps offset the lower contributions and less favorable rule evaluations associated with less egalitarian fairness views, its effects should be stronger among participants with less egalitarian pre-treatment fairness views. Accordingly, we estimated models that included an interaction between the vote-condition indicator and the pre-treatment egalitarian index. Under this prediction, the interaction coefficient should be negative. Table 8 presents the results.

Panel A reports the OLS results. In column (1), which includes no additional control variables, the interaction between voting condition and the egalitarian index was negative and statistically significant (*b* = −18.740, *SE* = 8.017, standardized *β* = −0.241, *p* = 0.021, 95% CI [−34.593, −2.886]), indicating that the estimated voting effect on contribution decreased as the egalitarian index increased. After controlling for individual characteristics in column (2), the interaction remained negative and statistically significant (*b* = −17.000, *SE* = 7.879, standardized *β* = −0.218, *p* = 0.033, 95% CI [−32.586, −1.414]). The same pattern emerged for rule acceptance. Without additional controls, the interaction term was negative and statistically significant (*b* = −3.634, *SE* = 1.796, standardized *β* = −0.174, *p* = 0.045, 95% CI [−7.185, −0.083]). After individual-characteristic controls were included, the interaction remained negative and statistically significant (*b* = −4.495, *SE* = 1.663, standardized *β* = −0.215, *p* = 0.008, 95% CI [−7.785, −1.204]). Panel B reports two-limit Tobit estimates for contribution and ordered logit estimates for rule acceptance. In the Tobit models for contribution, the interaction was negative and statistically significant both without additional controls (*b* = −29.155, *SE* = 10.580, *p* = 0.006, 95% CI [−49.891, −8.419]) and with controls for individual characteristics (*b* = −27.933, *SE* = 9.772, *p* = 0.004, 95% CI [−47.086, −8.780]). In the ordered logit models for rule acceptance, the interaction was negative and marginally significant without additional controls (*b* = −3.698, *SE* = 1.901, *p* = 0.052, 95% CI [−7.423, 0.028]) and statistically significant with controls for individual characteristics (*b* = −4.876, *SE* = 1.906, *p* = 0.011, 95% CI [−8.611, −1.140]). These results provide evidence that the effects of voting were larger among participants with less egalitarian pre-treatment fairness views.

**Figure 1:**
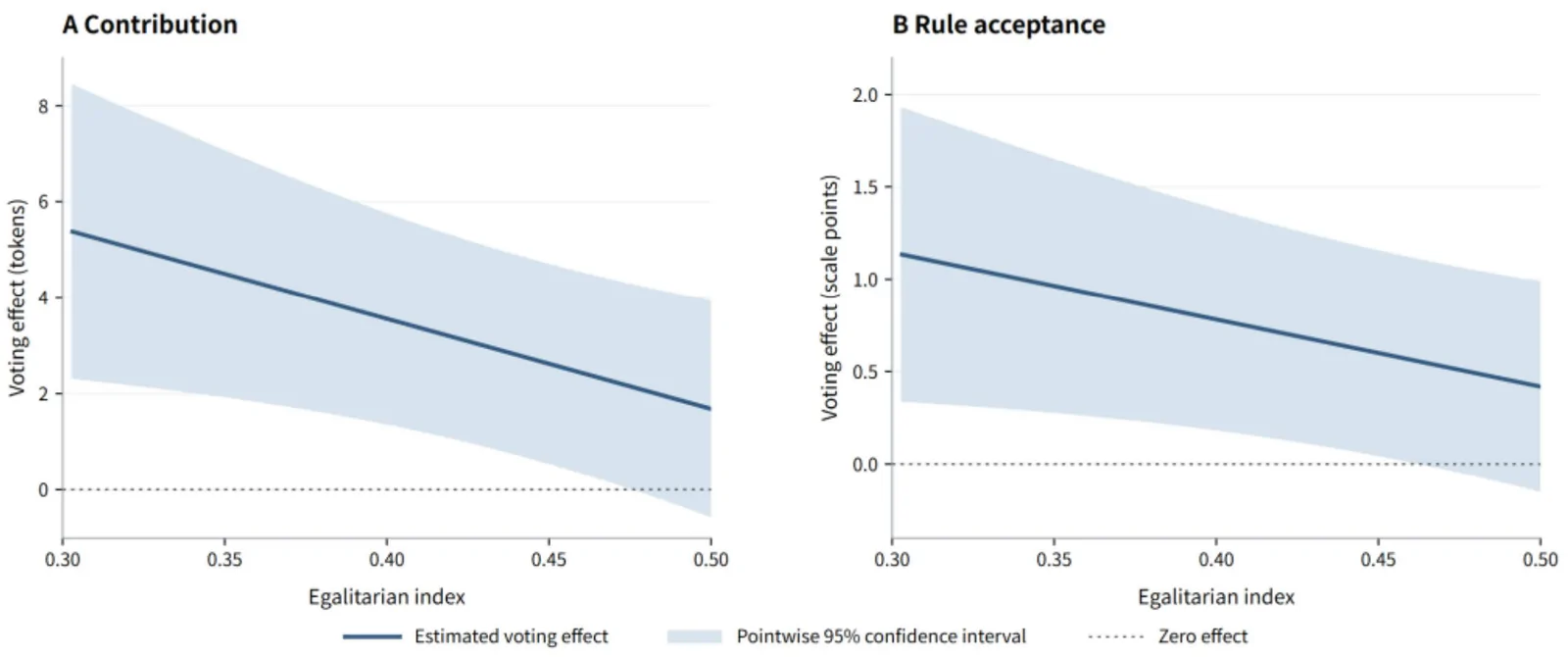
The marginal treatment effects of voting on contribution and rule acceptance by pre-treatment egalitarian index. Panel (**A**) shows the estimated effect on contribution, and Panel (**B**) shows the estimated effect on rule acceptance. Estimates are based on the OLS interaction models without additional controls in Table 8, Panel A, columns (1) and (3). Pointwise 95% confidence intervals are based on robust standard errors. The displayed index range is 0.303–0.500 (132 of 140 participants), but all 140 participants in the analytic sample were used to estimate the models.

To illustrate the interaction patterns, Figure 1 plots the estimated effect of voting on contribution and rule acceptance as a function of the pre-treatment egalitarian index, using the OLS specifications without additional controls in columns (1) and (3) of Table 8, Panel A. For visual clarity, the figure focuses on index values between 0.303 and 0.500, which contain 94.3% of the sample. The estimated voting effect declines as the egalitarian index increases for both outcomes. We further evaluated the simple effects of voting at the 25th and 75th percentiles of the pre-treatment egalitarian index, representing relatively low and high levels of egalitarianism within the sample. At the 25th percentile (0.394), voting increased contribution by an estimated 3.672 tokens (*SE* = 1.119, *p* = 0.001, 95% CI [1.459, 5.885]) and rule acceptance by 0.803 scale points (*SE* = 0.305, *p* = 0.009, 95% CI [0.200, 1.406]). At the 75th percentile (0.455), the corresponding effects were smaller but remained positive and statistically significant: 2.537 tokens for contribution (*SE* = 1.045, *p* = 0.017, 95% CI [0.470, 4.603]) and 0.583 scale points for rule acceptance (*SE* = 0.279, *p* = 0.038, 95% CI [0.032, 1.134]). These exploratory findings are consistent with the prediction that voting has larger positive effects among participants with less egalitarian pre-treatment fairness views.

Appendix B.3 reports supplementary analyses that include participants who failed the comprehension check and additionally control for expected peer contribution. Across these analyses, the estimated effects of voting on contribution and rule acceptance remained positive, while the interactions between voting and the egalitarian index remained negative. The interaction results were robust to controlling for expected peer contribution, although the evidence became weaker when the full sample was used. Overall, these analyses support the direction of the primary findings.

In summary, Experiment 3 shows that the procedure through which participation under the equal-sharing arrangement is determined can affect participants’ responses to the rule. When a group vote determined whether the group would participate under the equal-sharing rule, they contributed more and reported greater acceptance of the rule than when participation was imposed.^7^ Exploratory analyses provide suggestive evidence that the effects of voting were larger among participants with less egalitarian pre-treatment fairness views.

## 5. Discussion

In the context of an equal-sharing rule, this study examines whether egalitarian fairness views are associated with individual contributions in a team production setting, whether fairness-related prompting affects contributions, and how the procedure for deciding participation affects rule acceptance and contributions. We study these questions in a public goods game in which returns from the public account are shared equally among group members.

Across the first two experiments, we find that contributions under the equal-sharing rule are associated with measured fairness views and can be affected by fairness-related prompting. In Experiment 1, participants with more egalitarian fairness ideals contributed more to the public account. Experiment 2 provided causal evidence on the effect of fairness-related prompting by randomly assigning participants to generate reasons why equal sharing is either fair or unfair. Participants asked to generate reasons why equal sharing is fair subsequently contributed more than those asked to generate reasons why it is unfair.

These findings complement the incentive-centered perspective that is prominent in economic analyses of compensation and allocation rules. This perspective typically emphasizes the objective incentive properties of allocation rules, such as the link between individual effort and reward and the incentives for free riding. Our results suggest that contributions under equal sharing may also be related to how individuals evaluate the rule from a fairness perspective. The same allocation rule may therefore elicit different levels of contribution from individuals who hold different fairness ideals. Our study thus also extends existing evidence on heterogeneity in fairness ideals (e.g., Cappelen et al., 2007, 2010; Rodriguez-Lara & Moreno-Garrido, 2012; Luhan et al., 2019; Almås et al., 2020, 2025c) by showing that such heterogeneity is associated with contribution differences under an allocation rule.

One possible interpretation of the above findings is that they reflect the effects of the alignment between participants’ fairness ideals and the allocation rule. That is, individuals whose fairness views are more consistent with the allocation rule they face may respond more favorably to the rule and contribute more under it. In Experiment 1, participants with more egalitarian fairness views contributed more when returns from the public account were shared equally. In Experiment 2, prompting participants to consider why equal sharing is fair may have made the rule more consistent with their fairness views, potentially encouraging higher contributions. However, because the allocation rule itself was not varied, these experiments cannot determine whether the observed patterns reflect the alignment between participants’ fairness ideals and the allocation rule or a more general positive association between egalitarianism and cooperation. Identifying an alignment effect conclusively would require varying the allocation rule and testing the interaction between participants’ fairness ideals and the allocation rule.

An alternative interpretation is provided by the literature on moral preferences, which suggests that individuals are partly motivated to choose actions they perceive as morally appropriate (Tappin & Capraro, 2018; Bašić & Verrina, 2024). From this perspective, in Experiment 1, participants with more egalitarian fairness views may have been more likely to regard contributing under an equal-sharing rule as morally appropriate and consequently contributed more. Similarly, in Experiment 2, fairness-related prompting may have changed what level of contribution participants perceived as morally appropriate, thereby affecting subsequent contributions.

Experiment 3 in our study further highlights the role of the participation procedure. When participants were allowed to vote on whether their group would participate under the equal-sharing rule, rather than having participation imposed on them, they contributed more and rated the rule as fairer. Exploratory analyses provide suggestive evidence that the effects of voting were stronger among participants with less egalitarian pre-existing fairness views. These findings suggest that responses to the same allocation rule can be affected by the procedure through which participation under it is determined.

These findings are also consistent with the moral-preference perspective. By giving participants a role in determining whether the group would participate under the allocation arrangement, the voting procedure may have made the allocation arrangement seem more legitimate and cooperation under it more morally appropriate, potentially encouraging higher contributions. However, because the voting treatment bundles several factors—including voice, democratic choice, beliefs about others’ votes, and the possibility of receiving a fixed payoff—this interpretation should be viewed as one plausible mechanism rather than an independently identified causal channel.^8^

The pattern in Experiment 3 is also related to previous experimental studies comparing democratically or endogenously chosen institutions with exogenously imposed institutions in public goods settings (e.g., Tyran & Feld, 2006; Sutter et al., 2010; Gallier, 2020). Some of these studies find higher cooperation under endogenously chosen institutions (Tyran & Feld, 2006; Sutter et al., 2010), although Gallier (2020) shows that the apparent effect of democratic participation may partly reflect self-selection and information conveyed by voting. In particular, Vollan et al. (2017) show that responses to democratic versus imposed institutions can vary with individuals’ broader values. Our study complements Vollan et al. (2017) by shifting attention from general individual values to fairness views specifically relevant to the distributive rule under which participants interact.

The findings of this study point to two potential implications for the design and implementation of allocation arrangements in organizational settings. Organizations often evaluate allocation rules primarily in terms of their incentive properties, but our results suggest that individuals’ fairness evaluations of these rules may also matter for their willingness to contribute. Thus, when implementing a compensation or allocation rule, organizations may benefit from recognizing that individuals with different fairness views may respond differently to the same rule, rather than focusing solely on its material incentives. In addition, the findings from Experiment 3 suggest that organizations may improve the effectiveness of allocation arrangements by paying attention not only to the rule itself but also to the procedure used to determine participation under it. Allowing organizational members a meaningful role in the decision process and ensuring that the procedure is perceived as fair may strengthen their acceptance of the arrangement and encourage greater contribution. These implications should be interpreted cautiously, however, because our one-shot public goods experiments do not reproduce real-effort team production, employment relationships, repeated interactions, or organizational compensation systems. These implications should therefore be viewed as hypotheses for future real-effort and field research rather than as direct prescriptions for organizational compensation design.

Our study also has some limitations. The first limitation concerns the external validity and generalizability of our findings. All participants were students recruited from a single university and therefore constituted a relatively homogeneous sample. In addition, the one-shot public goods game abstracts from several important features of real-world team production, including real effort, employment relationships, repeated interactions, reputational concerns, and higher-stakes consequences. These features limit the external validity of our findings, particularly their generalizability to organizational settings and sustainability-relevant contexts such as resource-sharing communities. Future research using more diverse participant populations, real-effort tasks, repeated interactions, and field settings is needed to examine whether the observed patterns generalize beyond the present experimental setting.

A second limitation concerns the identification of the effects of the alignment between participants’ fairness ideals and the allocation rule. Because all three experiments implement the same equal-sharing rule, the present design cannot conclusively distinguish the effects of the alignment between participants’ fairness ideals and the allocation rule from a more general relationship between fairness views and cooperation. Future research could address this limitation by varying the allocation rule and examining its interaction with participants’ fairness views. In addition, the prompting task in Experiment 2 may have activated different norms, induced demand effects, or encouraged participants to behave consistently with what they had just written. The present design therefore cannot isolate the specific mechanism underlying the effect of the prompting. Moreover, because Experiment 2 does not include a neutral condition, it cannot determine whether the fair prompt increases contributions, the unfair prompt decreases them, or both.

A third limitation concerns the measurement of fairness views. Although our supplementary analyses in Appendix B.1.4 support the robustness of the abbreviated measure, the primary measure is based on two scenario-specific allocation judgments and therefore cannot fully capture the complexity or stability of individuals’ fairness ideals. Rule acceptance is also measured using a single, context-specific item. Future research could employ broader and repeated measures of fairness views and rule evaluations.

A fourth limitation concerns Experiment 3. Participants made their subsequent decisions under the assumption that their group had voted to participate, regardless of the realized voting outcome, and such conditional decisions may differ from choices made after an actual collective decision is known. Moreover, because the voting treatment combines several procedural and informational factors, the present design cannot isolate perceived procedural fairness as the mechanism underlying the observed effects. Future research could examine behavior after realized voting outcomes while avoiding selection concerns arising from conditioning on realized participation, and could more directly separate procedural fairness from other factors of the voting procedure.

## 6. Conclusions

Across three experiments using a public goods game, this paper examines whether individuals’ fairness views are associated with their contributions under an equal-sharing rule, whether fairness-related prompting changes contributions, and whether the procedure used to determine participation under the equal-sharing arrangement affects rule acceptance and contributions. Our findings indicate that participants with more egalitarian fairness views contributed more under the rule, while prompting participants to generate reasons why equal sharing is fair, rather than unfair, led to higher subsequent contributions. When participation was determined through a group vote rather than imposed, participants contributed more and evaluated the equal-sharing rule more favorably. Exploratory evidence further indicates that the effects of voting may be stronger among participants with less egalitarian fairness views. Taken together, these findings highlight the relevance of fairness-related evaluations and participation procedures for understanding behavior under equal-sharing arrangements.

## Figures and Tables

**Table 1:** Production components in Question 1.

Person	Talent	Effort	Luck	Total
A	5	8	0	13
B	12	5	3	20

**Table 2:** Production components in Question 2.

Person	Talent	Effort	Luck	Total
A	8	5	3	16
B	5	12	0	17

**Table 3:** Allocation options in Question 2.

Person	Option 1	Option 2	Option 3	Option 4
A	16	14.5	13	16.5
B	17	18.5	20	16.5

**Table 4:** Association between egalitarian index and contributions.

	(1) Contribution OLS	(2) Contribution OLS	(3) Contribution Tobit	(4) Contribution Tobit
Egalitarian index	11.686 *** (1.794)	12.331 *** (2.022)	17.916 *** (3.910)	19.012 *** (4.146)
Individual characteristics	No	Yes	No	Yes
Constant	5.889 *** (1.164)	8.615 ** (3.612)	3.793 * (2.001)	10.824 ** (5.198)
*N*	123	123	123	123
*R*^2^/Pseudo *R*^2^	0.140	0.170	0.027	0.036

Note. Robust standard errors are reported in parentheses. Individual-characteristic controls include risk preference, gender, age, family location, and household per capita monthly income. * *p* < 0.10; ** *p* < 0.05; *** *p* < 0.01.

**Table 5:** Effect of fairness prompting on contributions.

	(1) Contribution OLS	(2) Contribution OLS	(3) Contribution Tobit	(4) Contribution Tobit
Fair-prompt condition	2.221 *** (0.787)	1.829 ** (0.777)	3.522 *** (1.184)	2.960 *** (1.145)
Individual characteristics	No	Yes	No	Yes
Constant	9.652 *** (0.494)	−1.369 (3.416)	9.430 *** (0.693)	−7.603 (5.440)
*N*	250	250	250	250
*R*^2^/Pseudo *R*^2^	0.032	0.116	0.007	0.024

Note. Robust standard errors are reported in parentheses. Fair-prompt condition equals 1 for participants assigned to the fair-prompt condition and 0 for those assigned to the unfair-prompt condition. Individual characteristics include risk preference, gender, age, household income category, and urban family location. ** *p* < 0.05; *** *p* < 0.01.

**Table 6:** Effect of voting on contributions.

	(1) Contribution OLS	(2) Contribution OLS	(3) Contribution Tobit	(4) Contribution Tobit
Vote condition	2.716 ** (1.054)	1.928 * (1.070)	4.441 *** (1.632)	3.255 ** (1.572)
Individual characteristics	No	Yes	No	Yes
Constant	10.345 *** (0.773)	−2.036 (4.270)	10.532 *** (1.127)	−8.763 (6.788)
*N*	140	140	140	140
*R*^2^/Pseudo *R*^2^	0.044	0.143	0.010	0.030

Note. Robust standard errors are reported in parentheses. Voting condition equals 1 for the vote condition and 0 for the no-vote condition. Individual-characteristic controls include risk preference, gender, age, household income category, and urban family location. * *p* < 0.10; ** *p* < 0.05; *** *p* < 0.01.

**Table 7:** Effect of voting on rule acceptance.

	(1) Rule Acceptance OLS	(2) Rule Acceptance OLS	(3) Rule Acceptance Ordered Logit	(4) Rule Acceptance Ordered Logit
Vote condition	0.612 ** (0.286)	0.518 * (0.282)	0.559 * (0.303)	0.539 * (0.305)
Individual characteristics	No	Yes	No	Yes
Constant	3.534 *** (0.212)	0.106 (1.316)	—	—
*N*	140	140	140	140
*R*^2^/Pseudo *R*^2^	0.031	0.120	0.007	0.040

Note. Robust standard errors are reported in parentheses. Voting condition equals 1 for the vote condition and 0 for the no-vote condition. Individual-characteristic controls include risk preference, gender, age, household income category, and urban family location. * *p* < 0.10; ** *p* < 0.05; *** *p* < 0.01.

**Table 8:** Interaction between voting and pre-treatment egalitarian views.

	(1) Contribution	(2) Contribution	(3) Rule Acceptance	(4) Rule Acceptance
Panel A. OLS				
Vote condition	2.806 *** (1.043)	2.071 * (1.072)	0.635 ** (0.281)	0.562 ** (0.272)
Egalitarian index (centered)	12.052 *** (3.431)	10.091 *** (2.980)	2.626 * (1.396)	2.886 ** (1.235)
Vote condition × egalitarian index	−18.740 ** (8.017)	−17.000 ** (7.879)	−3.634 ** (1.796)	−4.495 *** (1.663)
Individual characteristics	No	Yes	No	Yes
Constant	10.197 *** (0.753)	−2.467 (4.241)	3.502 *** (0.207)	0.010 (1.346)
*N*	140	140	140	140
*R* ^2^	0.086	0.173	0.056	0.151
Panel B. Tobit and ordered logit				
Vote condition	4.493 *** (1.585)	3.373 ** (1.536)	0.599 ** (0.301)	0.610 ** (0.301)
Egalitarian index (centered)	18.614 ** (7.323)	16.440 *** (6.034)	2.850 * (1.536)	3.535 ** (1.525)
Vote condition × egalitarian index	−29.155 *** (10.580)	−27.933 *** (9.772)	−3.698 * (1.901)	−4.876 ** (1.906)
Individual characteristics	No	Yes	No	Yes
Constant	10.356 *** (1.087)	−9.945 (6.714)	—	—
*N*	140	140	140	140
Pseudo *R*^2^	0.018	0.037	0.014	0.051

Note. Robust standard errors are reported in parentheses. Voting condition equals 1 for voting and 0 otherwise. The egalitarian index is centered at its pooled mean. Individual-characteristic controls include risk preference, gender, age, household income category, and urban family location. * *p* < 0.10; ** *p* < 0.05; *** *p* < 0.01.

## Data Availability

The data and analysis code supporting the findings of this study are publicly available on the Open Science Framework (OSF) at https://osf.io/kf5tm/overview?view_only=bef5422bb02541d097ed12b94900c146 (accessed on 20 September 2026).

## Notes

1 None of the three experiments reported in this article or their analysis plans were preregistered.

2 Our use of this abbreviated measure was informed by the robustness evidence reported by Luhan et al. (2019), who examined whether participants’ fairness ideals changed when they moved from the position of impartial spectators to that of stakeholders in a production and bargaining setting. In their study, participants completed the full seven-item questionnaire before the experiment and an abbreviated two-item version after the experiment. To assess whether their findings were driven by the different numbers of items used at the two stages, they reconstructed the pre-experiment measure using the same two items included in the post-experiment elicitation. The resulting analysis yielded substantively similar conclusions. This robustness finding provided support for our use of the abbreviated elicitation.

3 Because expected peer contribution was elicited after participants had made their own contribution decision, it may partly reflect projection or ex post rationalization and cannot be treated as a standard exogenous control variable. Accordingly, throughout Experiments 1–3, we exclude this measure from all primary regression specifications reported in the main text. For each experiment, specifications that additionally control for expected peer contribution are reported only in the Appendix B as supplementary robustness analyses. These specifications are presented only to assess whether the results are sensitive to including the belief measure and are not treated as the preferred estimates.

4 Across all experiments, we used a time-based stopping rule. Before recruitment began, we specified a closing date and time for each questionnaire, and data collection ended at that predetermined time. The resulting sample size was therefore determined by the number of participants who completed the experiment before the deadline. Data for Experiment 1 were collected in two waves. Wave 1 included 101 participants and used the abbreviated two-item fairness-ideal elicitation. Of these participants, 26 failed the comprehension test, leaving an analytic sample of 75. To assess the robustness of our findings to replacing the abbreviated measure with the full seven-item measure, we subsequently collected a second wave of data. Wave 2 comprised 59 participants, who completed the full seven-item fairness-ideal elicitation developed by Luhan et al. (2019). 11 participants failed the comprehension test, leaving an analytic sample of 48. Apart from the fairness-ideal elicitation, the contribution task and all other experimental procedures were identical across the two waves. The two questions used in Wave 1 corresponded to Question 1 and Question 6 of the full seven-item elicitation, allowing us to construct the same two-item index for both waves. Accordingly, our primary analyses pool the two waves and use this common two-item index. Supplementary analyses using the full seven-item index for the Wave 2 sample are reported in Appendix B.1.4.

5 Before receiving any condition-specific instructions, each participant selected one of two visually identical options. Each option was linked to one condition, and Wenjuanxing randomized the display positions of the two options separately for each participant. The selected option directed the participant to its associated condition.

6 To prevent repeated participation, each experiment was assigned a unique code that was displayed in its recruitment announcement. Participants were informed that they could participate only once under the same experiment code. Recruitment announcements for Experiment 2 also listed the code of Experiment 1 and stated that students who had participated in Experiment 1 were ineligible. In addition, each Wenjuanxing link was configured to allow for only one submission per device and per account. After data collection, we cross-checked the Alipay account identifiers provided for payment within and across all experimental samples. We found no evidence of duplicate participation.

7 Among the 82 participants in the analytic sample assigned to the vote condition, 71 (86.6%) voted in favor of participation and 11 (13.4%) voted against it. Because only 11 participants voted against participation, analyses separated by participants’ own votes would be substantially underpowered and likely to yield unstable estimates. We therefore do not report subgroup analyses based on participants’ own votes.

8 To examine whether nonbinding voice alone could account for the voting effect, we conducted an additional condition in which participants could express their preference regarding participation, but their expressed preference did not affect whether the public goods arrangement was implemented. Contributions in this nonbinding-voice condition did not differ from those in the no-vote condition and were lower than those in the vote condition. This supplementary evidence suggests that the opportunity to express a nonbinding opinion alone does not account for the contribution effect of voting. Appendix C provides details on the design and sample of the additional condition and reports the corresponding descriptive statistics and regression results.

## Abbreviations

The following abbreviations are used in this manuscript:

OLS	Ordinary least squares
CNY	Chinese yuan

## Appendix A. Experimental Instructions (Translated from Chinese)

### Appendix A.1. Experiment 1

#### Appendix A.1.1. General Information

Thank you for your participation in this experiment! Please read the following instructions carefully. You will receive earnings for participating in this experiment. The amount will depend on your decisions and the decisions of others. At the end, you will be tested on your understanding of the experimental rules. You will receive experimental earnings only if you understand the rules, so please read them carefully. Earnings are expressed in experimental tokens. Tokens will be converted into Chinese yuan at a rate of 10 tokens = CNY 1. In addition to any experimental earnings, you will receive a fixed participation fee of CNY 1. Your total payment will be transferred to the Alipay account you provide. There are no right or wrong answers. Please give your honest opinion. Your behavior and responses will remain anonymous throughout the experiment.

#### Appendix A.1.2. Fairness-Ideal Elicitation^9^

Imagine the following situation: two individuals, A and B, have jointly produced an output. Each has contributed to this joint output. Each person’s contribution comes from talent, effort, and luck, and the contributions from these sources can be expressed numerically.

**Question 1**

Suppose that A and B’s contributions to the joint output are as follows:

**Person**	**Talent**	**Effort**	**Luck**	**Total**
A	5	8	0	13
B	12	5	3	20

The total value of the joint output to be allocated is 33. How do you think this output should be fairly allocated between A and B?

A receives: ______ B receives: ______

**Question 2**

Suppose that A and B’s contributions to the joint output are as follows:

**Person**	**Talent**	**Effort**	**Luck**	**Total**
A	8	5	3	16
B	5	12	0	17

The total value of the joint output to be allocated is 33. Select the allocation you consider fairest. Please give your honest opinion.

**Person**	**Option 1**	**Option 2**	**Option 3**	**Option 4**
A	16	14.5	13	16.5
B	17	18.5	20	16.5

#### Appendix A.1.3. Public Goods Game

You will be randomly grouped with three other participants. The other three group members are real participants who will also make decisions. All four of you will read the following instructions.

First, each of you will receive 20 experimental tokens. Next, each of you must decide how many of these 20 tokens to keep and how many to contribute to a public pool. You may contribute between 0 and 20 tokens. All tokens contributed to the public pool will be doubled and then divided equally among the four of you. Your earnings from this task will equal the tokens you keep plus the tokens you receive from the public pool.

For example:

If each person contributes 20 tokens, the group contributes 80 tokens in total. The public pool doubles these tokens, so the group receives 160 tokens, divided equally among the four members. Each person therefore receives 40 tokens.

If you keep all 20 tokens and the other three members each contribute 20 tokens, the group contributes 60 tokens in total. The public pool doubles these tokens, so the group receives 120 tokens, divided equally among the four members. You receive 50 tokens: 30 from the public pool plus the 20 you kept. Each of the other members receives 30 tokens.

If you and all other group members keep all 20 tokens, none of you receives any additional tokens. Each person finishes with the initial 20 tokens.

**Rule acceptance**

To what extent do you think it is fair to divide the funds in the public pool equally among all group members?

Select one response: 1, 2, 3, 4, 5, 6, or 7. 1 = Very unfair; 7 = Very fair.

**Contribution**

Enter the number of experimental tokens you would like to contribute to the public pool: ______

**Expected peer contribution**

How many tokens do you predict the other three members will contribute on average? ______

**Comprehension questions**

How many tokens should you contribute to the public pool to maximize the earnings of the whole group? ______

How many tokens should you contribute to the public pool to maximize your own earnings? ______

#### Appendix A.1.4. Risk Preference and Demographic Information

Generally speaking, are you a person who is very willing to take risks, or do you try to avoid taking risks? Select one response from 1 to 10 (1 = Very unwilling to take risks; 10 = Very willing to take risks).

Gender: Male/Female.

Age: ______

Family residence: Urban/Rural.

Monthly household income per person: Below CNY 2000; CNY 2000–4000; CNY 4000–6000; CNY 6000–8000; above CNY 8000.

### Appendix A.2. Experiment 2

#### Appendix A.2.1. General Information

*The general information was identical to* *Appendix A.1.1.*

#### Appendix A.2.2. Fairness-Related Writing Task

*Participants received the prompt corresponding to their assigned condition.*

**Unfair-prompt condition**

Please give some reasons supporting the view that “equal sharing is unfair.”

Your response: __________________________________________________

**Fair-prompt condition**

Please give some reasons supporting the view that “equal sharing is fair.”

Your response: __________________________________________________

#### Appendix A.2.3. Fairness-Ideal Elicitation

*After the writing task, participants answered the same two fairness-ideal elicitation questions as in Appendix A.1.2, with the same numerical tables and response options.*

#### Appendix A.2.4. Public Goods Game

*The game instructions were identical to Appendix A.1.3.*

#### Appendix A.2.5. Risk Preference and Demographic Information

*The questions and response options were identical to Appendix A.1.4.*

### Appendix A.3. Experiment 3

#### Appendix A.3.1. General Information

*The general information was identical to Appendix A.1.1.*

#### Appendix A.3.2. Fairness-Ideal Elicitation

*Participants answered the same two fairness-ideal elicitation questions as in Appendix A.1.2, with the same numerical tables and response options.*

#### Appendix A.3.3. Public Goods Game

**No-vote condition**

*The game instructions were identical to Appendix A.1.3. Participants then proceeded directly to Appendix A.3.4.*

**Vote condition**

You will be randomly grouped with three other participants. The other three group members are real participants who will also make decisions. All four of you will read the following instructions.

First, each of you will receive 20 experimental tokens. Next, you will vote on whether to take part in a group interaction. The decision will be made by majority vote; if the votes are tied, the computer will choose randomly. If the group does not take part, each of you will keep your own 20 tokens. If the group takes part, the interaction rules are as follows:

*At this point, participants received the same contribution rules and all three numerical examples reproduced in Appendix A.1.3, from “Next, each of you must decide how many of these 20 tokens to keep” through the example in which everyone keeps all 20 tokens.*

Please vote on whether to take part in the group interaction.

Select one option: Take part/Do not take part.

Assume that your group’s final vote is to take part in the interaction. Please answer the following questions. If the vote is to take part, we will calculate your payment according to your answers.

*Participants then answered the same questions, in the same order, as in Appendix A.1.3.*

#### Appendix A.3.4. Risk Preference and Demographic Information

*The questions and response options were identical to Appendix A.1.4.*

## Appendix B. Supplementary Robustness Analyses

### Appendix B.1. Supplementary Robustness Analyses for Experiment 1

#### Appendix B.1.1. Including Participants Who Failed the Comprehension Check

Because participants completed the comprehension check after making their contribution decisions, we re-estimated the main models using all 160 participants who completed Experiment 1, including those who failed the comprehension check. As shown in Table A1, the OLS models indicated a positive and statistically significant association between the egalitarian index and contribution both without additional controls (*b* = 9.169, *SE* = 2.161, standardized *β* = 0.285, *p* < 0.001, 95% CI [4.900, 13.437]) and with controls for individual characteristics (*b* = 10.034, *SE* = 2.257, standardized *β* = 0.311, *p* < 0.001, 95% CI [5.576, 14.492]). The two-limit Tobit models yielded similar results, both without controls (*b* = 13.897, *SE* = 3.987, *p* < 0.001, 95% CI [6.082, 21.711]) and with controls for individual characteristics (*b* = 15.213, *SE* = 4.040, *p* < 0.001, 95% CI [7.295, 23.131]). These results indicate that the conclusions from Experiment 1 are robust to including participants who failed the comprehension check.

**Table A1:** The association between the egalitarian index and contributions in the full sample.

	(1) Contribution OLS	(2) Contribution OLS	(3) Contribution Tobit	(4) Contribution Tobit
Egalitarian index	9.169 *** (2.161)	10.034 *** (2.257)	13.897 *** (3.987)	15.213 *** (4.040)
Individual characteristics	No	Yes	No	Yes
Constant	7.157 *** (1.174)	7.244 ** (3.331)	5.833 *** (1.951)	8.254 * (5.002)
*N*	160	160	160	160
*R*^2^/Pseudo *R*^2^	0.081	0.119	0.015	0.023

Note. The sample includes all 160 participants who completed Experiment 1, including the 37 participants who failed the comprehension check. Individual-characteristic controls include risk preference, gender, age, family location, and household per capita monthly income. Robust standard errors are reported in parentheses. The Tobit models are left-censored at 0 and right-censored at 20. * *p* < 0.10; ** *p* < 0.05; *** *p* < 0.01.

#### Appendix B.1.2. Controlling for Expected Peer Contribution

Expected peer contribution was elicited after participants had made their own contribution decisions. It could therefore reflect participants’ projection of their own behavior onto their peers or an ex post rationalization of their contribution decisions, rather than an independently measured prior expectation. We therefore excluded this variable from the primary specifications and included it only in the supplementary robustness analyses reported in Table A2. Both models also include controls for individual characteristics. The association between the egalitarian index and contribution remained positive and statistically significant in both the OLS specification (*b* = 11.319, *SE* = 2.249, standardized *β* = 0.363, *p* < 0.001, 95% CI [6.864, 15.774]) and the two-limit Tobit specification (*b* = 17.564, *SE* = 4.386, *p* < 0.001, 95% CI [8.966, 26.161]). These results indicate that the relationship between the egalitarian index and contribution is robust to additionally controlling for expected peer contribution.

**Table A2:** The association between the egalitarian index and contributions after controlling for expected peer contribution.

	(1) Contribution OLS	(2) Contribution Tobit
Egalitarian index	11.319 *** (2.249)	17.564 *** (4.386)
Individual characteristics	Yes	Yes
Expected peer contribution	Yes	Yes
Constant	5.064 (4.114)	5.902 (5.685)
*N*	123	123
*R*^2^/Pseudo *R*^2^	0.202	0.042

Note. Robust standard errors are reported in parentheses. Individual-characteristic controls include risk preference, gender, age, family location, and household per capita monthly income. The Tobit models are left-censored at 0 and right-censored at 20. *** *p* < 0.01.

#### Appendix B.1.3. Using Rule Acceptance as an Alternative Measure

As a robustness check, we re-estimated the main models using rule acceptance as a context-specific measure of participants’ evaluation of the equal-sharing rule. As shown in Table A3, a one-unit increase in rule acceptance was associated with 1.676 additional contribution tokens without additional controls (*b* = 1.676, *SE* = 0.246, standardized *β* = 0.509, *p* < 0.001, 95% CI [1.189, 2.163]) and 1.919 additional tokens after the controls were included (*b* = 1.919, *SE* = 0.232, standardized *β* = 0.583, *p* < 0.001, 95% CI [1.460, 2.378]). The corresponding Tobit estimates were also positive and statistically significant both without additional controls (*b* = 2.194, *SE* = 0.387, *p* < 0.001, 95% CI [1.435, 2.953]) and with controls for individual characteristics (*b* = 2.626, *SE* = 0.392, *p* < 0.001, 95% CI [1.857, 3.394]). These results provide further evidence that more favorable fairness evaluations of the equal-sharing rule are associated with higher contributions.

**Table A3:** Association between rule acceptance and contributions in Experiment 1.

	(1) Contribution OLS	(2) Contribution OLS	(3) Contribution Tobit	(4) Contribution Tobit
Rule acceptance	1.676 *** (0.246)	1.919 *** (0.232)	2.194 *** (0.387)	2.626 *** (0.392)
Individual characteristics	No	Yes	No	Yes
Constant	4.261 *** (1.178)	11.434 *** (3.791)	2.690 (1.787)	14.762 *** (5.406)
*N*	123	123	123	123
*R*^2^/Pseudo *R*^2^	0.259	0.340	0.043	0.068

Note. Robust standard errors are reported in parentheses. Individual-characteristic controls include risk preference, gender, age, family location, and household per capita monthly income. The Tobit models are left-censored at 0 and right-censored at 20. *** *p* < 0.01.

#### Appendix B.1.4. Robustness of the Egalitarian Index Measurement

We first examined whether the primary findings depended on constructing the abbreviated egalitarian index as an equal-weight average of its two components. Among the 123 participants included in the primary analysis of Experiment 1, the open-response component had a mean of 0.806 (*SD* = 0.116), a median of 0.788, and a range of [0.121, 1.000]. For the multiple-choice component, 14.6% selected the egalitarian option, 8.9% selected the meritocratic option, 8.1% selected the choice-egalitarian option, and 68.3% selected the libertarian option. For constructing the multiple-choice component, the egalitarian option was coded as 1 and the other three options as 0. The two components were positively correlated (Pearson’s *r* = 0.284, *p* = 0.001, 95% CI [0.113, 0.439]; Spearman’s *ρ* = 0.376, *p* < 0.001).

Table A4 reports the results of models estimated using each component separately. The open-response component was positively and statistically significantly associated with contribution in the OLS model without additional controls (*b* = 11.738, *SE* = 5.603, standardized *β* = 0.216, *p* = 0.038, 95% CI [0.645, 22.830]). The relationship remained positive and statistically significant after controls for individual characteristics were added (*b* = 13.185, *SE* = 6.009, standardized *β* = 0.243, *p* = 0.030, 95% CI [1.283, 25.087]). The corresponding Tobit estimates were also positive without additional controls (*b* = 19.235, *SE* = 9.474, *p* = 0.042, 95% CI [0.667, 37.802]) and with controls for individual characteristics (*b* = 20.260, *SE* = 9.734, *p* = 0.037, 95% CI [1.182, 39.337]).

The multiple-choice component was likewise positively associated with contribution in the OLS specifications, both without additional controls (*b* = 6.300, *SE* = 1.062, standardized *β* = 0.356, *p* < 0.001, 95% CI [4.198, 8.402]) and with individual-characteristic controls (*b* = 6.647, *SE* = 1.183, standardized *β* = 0.375, *p* < 0.001, 95% CI [4.304, 8.991]). The corresponding Tobit estimates were also positive without additional controls (*b* = 9.304, *SE* = 2.222, *p* < 0.001, 95% CI [4.949, 13.658]) and with individual-characteristic controls (*b* = 10.038, *SE* = 2.376, *p* < 0.001, 95% CI [5.382, 14.694]).

These results indicate that the positive relationship with contribution emerged for each component and was not driven by the averaging rule used to construct the two-item index. In addition, because the data from Experiment 1 were collected in two waves, we re-estimated the primary models with an indicator for the data collection wave. The coefficients on the egalitarian index and rule acceptance remained positive and statistically significant in all OLS and Tobit specifications, both with and without individual-characteristic controls. Because space is limited, the detailed results are not reported.

**Table A4:** Contribution regressions using the two components of the abbreviated egalitarian index separately.

	(1) Contribution	(2) Contribution	(3) Contribution	(4) Contribution
Panel A. OLS				
Open-response component	11.738 ** (5.603)	13.185 ** (6.009)		
Multiple-choice component			6.300 *** (1.062)	6.647 *** (1.183)
Individual characteristics	No	Yes	No	Yes
Constant	1.991 (4.604)	1.328 (6.555)	10.533 *** (0.605)	14.706 *** (3.593)
*N*	123	123	123	123
*R* ^2^	0.047	0.076	0.126	0.155
Panel B. Tobit				
Open-response component	19.235 ** (9.474)	20.260 ** (9.734)		
Multiple-choice component			9.304 *** (2.222)	10.038 *** (2.376)
Individual characteristics	No	Yes	No	Yes
Constant	−3.197 (7.672)	−0.792 (9.988)	10.941 *** (0.874)	20.252 *** (5.455)
*N*	123	123	123	123
Pseudo *R*^2^	0.010	0.017	0.023	0.032

Note. Columns (1) and (2) use the open-response component; columns (3) and (4) use the multiple-choice component. Robust standard errors are reported in parentheses. Individual-characteristic controls include risk preference, gender, age, family location, and household per capita monthly income. The Tobit models are left-censored at 0 and right-censored at 20. ** *p* < 0.05; *** *p* < 0.01.

As a further assessment of measurement robustness, we examined whether the results based on the abbreviated two-item index were consistent with those obtained using the complete seven-item fairness-ideal elicitation developed by Luhan et al. (2019). Participants in the second data collection wave completed all seven items, while all other experimental procedures were identical to those used in the first wave. Among the 48 participants who passed the comprehension check, the seven-item index had good internal consistency (Cronbach’s *α* = 0.805). The abbreviated two-item index and the seven-item index were strongly correlated (Pearson’s *r* = 0.821, *p* < 0.001, 95% CI [0.701, 0.896]; Spearman’s *ρ* = 0.620, *p* < 0.001). The two-item index had a mean of 0.466 (*SD* = 0.174; median = 0.394; range [0.303, 1.000]), and the seven-item index had a mean of 0.467 (*SD* = 0.145; median = 0.407; range [0.354, 1.000]).

Table A5 presents the results obtained after replacing the abbreviated two-item index with the complete seven-item index. The seven-item index was positively associated with contribution without additional controls in OLS (*b* = 12.593, *SE* = 3.329, standardized *β* = 0.315, *p* < 0.001, 95% CI [5.893, 19.293]) and Tobit models (*b* = 19.716, *SE* = 7.582, *p* = 0.009, 95% CI [4.528, 34.577]). The estimates remained positive after adding individual-characteristic controls in OLS (*b* = 13.653, *SE* = 5.248, standardized *β* = 0.341, *p* = 0.013, 95% CI [3.054, 24.253]) and Tobit models (*b* = 22.344, *SE* = 9.620, *p* = 0.020, 95% CI [3.489, 41.200]). These results indicate that the relationship between egalitarian fairness views and contribution is robust to using the complete seven-item measure instead of the abbreviated two-item measure.

**Table A5:** The association between the seven-item egalitarian index and contributions.

	(1) Contribution OLS	(2) Contribution OLS	(3) Contribution Tobit	(4) Contribution Tobit
Seven-item egalitarian index	12.593 *** (3.329)	13.653 ** (5.248)	19.716 *** (7.582)	22.344 ** (9.620)
Individual characteristics	No	Yes	No	Yes
Constant	5.496 *** (2.001)	14.452 (10.084)	2.787 (3.701)	16.346 (13.550)
*N*	48	48	48	48
*R*^2^/Pseudo *R*^2^	0.099	0.277	0.020	0.061

Note. Each column reports a separate regression using participants in the second data collection wave who passed the comprehension check. Robust standard errors are reported in parentheses. Individual-characteristic controls include risk preference, gender, age, family location, and household per capita monthly income. The Tobit models are left-censored at 0 and right-censored at 20. ** *p* < 0.05; *** *p* < 0.01.

### Appendix B.2. Supplementary Robustness Analyses for Experiment 2

#### Appendix B.2.1. Including Participants Who Failed the Comprehension Check

We repeated the primary analyses using all 332 initially recruited participants: 165 in the fair-prompt condition and 167 in the unfair-prompt condition. Mean contribution was 11.733 tokens (*SE* = 0.532) in the fair-prompt condition and 10.024 tokens (*SE* = 0.457) in the unfair-prompt condition. The Mann–Whitney U test indicated that this difference was statistically significant (*p* = 0.012).

Table A6 reports the corresponding regression estimates. In the OLS model without additional controls, the estimated treatment effect was positive and statistically significant (*b* = 1.709, *SE* = 0.701, standardized *β* = 0.133, *p* = 0.015, 95% CI [0.330, 3.089]). The estimate remained positive and statistically significant after controls for individual characteristics were added (*b* = 1.486, *SE* = 0.688, standardized *β* = 0.116, *p* = 0.031, 95% CI [0.133, 2.838]). The two-limit Tobit models yielded consistent results, both without additional controls (*b* = 2.840, *SE* = 1.053, *p* = 0.007, 95% CI [0.776, 4.903]) and with controls for individual characteristics (*b* = 2.504, *SE* = 1.018, *p* = 0.014, 95% CI [0.508, 4.499]). These results indicate that the treatment effect remains when participants who failed the comprehension check are included.

**Table A6:** Effect of fairness prompting on contributions in full sample.

	(1) Contribution OLS	(2) Contribution OLS	(3) Contribution Tobit	(4) Contribution Tobit
Fair-prompt condition	1.709 ** (0.701)	1.486 ** (0.688)	2.840 *** (1.053)	2.504 ** (1.018)
Individual characteristics	No	Yes	No	Yes
Constant	10.024 *** (0.457)	2.403 (3.171)	10.054 *** (0.642)	−2.089 (4.908)
*N*	332	332	332	332
*R*^2^/Pseudo *R*^2^	0.018	0.086	0.004	0.018

Note. The sample includes all 332 participants who completed Experiment 2, including the 82 participants who failed the comprehension check. Fair-prompt condition equals 1 for participants assigned to the fair-prompt condition and 0 for those assigned to the unfair-prompt condition. Robust standard errors are reported in parentheses. Individual-characteristic controls include risk preference, gender, age, family location, and household per capita monthly income. The Tobit models are left-censored at 0 and right-censored at 20. ** *p* < 0.05; *** *p* < 0.01.

#### Appendix B.2.2. Controlling for Expected Peer Contribution

Table A7 reports models that additionally control for expected peer contribution. The estimated difference between the two prompting conditions remained positive and was marginally significant at the 10% level in the OLS model (*b* = 1.299, *SE* = 0.731, standardized *β* = 0.104, *p* = 0.077, 95% CI [−0.140, 2.738]). In the two-limit Tobit model, the estimated difference was positive and statistically significant (*b* = 2.285, *SE* = 1.048, *p* = 0.029, 95% CI [0.230, 4.339]). Thus, the supplementary models continued to indicate higher contributions in the fair-prompt condition after controlling for expected peer contribution.

**Table A7:** Effect of fairness prompting on contributions after controlling for expected peer contribution.

	(1) Contribution OLS	(2) Contribution Tobit
Fair-prompt condition	1.299 * (0.731)	2.285 ** (1.048)
Individual characteristics	Yes	Yes
Expected peer contribution	Yes	Yes
Constant	−4.158 (2.692)	−11.828 *** (4.517)
*N*	250	250
*R*^2^/Pseudo *R*^2^	0.297	0.064

Note. Both models control for expected peer contribution, risk preference, gender, age, family location, and household income category. Fair-prompt condition equals 1 for participants assigned to the fair-prompt condition and 0 for those assigned to the unfair-prompt condition. Robust standard errors are reported in parentheses. The Tobit model is left-censored at 0 and right-censored at 20. * *p* < 0.10; ** *p* < 0.05; *** *p* < 0.01.

### Appendix B.3. Supplementary Robustness Analyses for Experiment 3

#### Appendix B.3.1. Including Participants Who Failed the Comprehension Check

We repeated the contribution analyses using all 208 participants who completed Experiment 3, including 118 in the vote condition and 90 in the no-vote condition. The mean contribution was 12.958 tokens (*SE* = 0.552) in the vote condition and 10.911 tokens (*SE* = 0.645) in the no-vote condition. The Mann–Whitney U test indicated that this difference was statistically significant (*p* = 0.019).

Table A8 reports the corresponding full-sample regression estimates. In column (1), the unadjusted OLS estimate was positive and statistically significant (*b* = 2.047, *SE* = 0.849, standardized *β* = 0.166, *p* = 0.017, 95% CI [0.373, 3.720]). After controls for individual characteristics were added in column (2), the estimate remained positive and statistically significant (*b* = 1.921, *SE* = 0.825, standardized *β* = 0.156, *p* = 0.021, 95% CI [0.295, 3.548]). The two-limit Tobit estimates were likewise positive and statistically significant both without additional controls in column (3) (*b* = 3.156, *SE* = 1.268, *p* = 0.013, 95% CI [0.671, 5.641]) and with controls for individual characteristics in column (4) (*b* = 2.991, *SE* = 1.205, *p* = 0.013, 95% CI [0.629, 5.352]). Thus, the positive effect of voting on contribution is robust to including participants who failed the comprehension check.

**Table A8:** Effect of voting on contributions in full sample.

	(1) Contribution OLS	(2) Contribution OLS	(3) Contribution Tobit	(4) Contribution Tobit
Vote condition	2.047 ** (0.849)	1.921 ** (0.825)	3.156 ** (1.268)	2.991 ** (1.205)
Individual characteristics	No	Yes	No	Yes
Constant	10.911 *** (0.645)	−1.411 (3.420)	11.418 *** (0.929)	−6.919 (5.403)
*N*	208	208	208	208
*R*^2^/Pseudo *R*^2^	0.028	0.131	0.006	0.026

Note. The sample includes all 208 participants who completed Experiment 3, including the 68 participants who failed the comprehension check. The vote condition is coded as 1, with the no-vote condition serving as the reference category. Robust standard errors are reported in parentheses. Individual-characteristic controls include risk preference, gender, age, family location, and household per capita monthly income. The Tobit models are left-censored at 0 and right-censored at 20. ** *p* < 0.05; *** *p* < 0.01.

We repeated the analyses of rule acceptance using all 208 participants initially recruited for Experiment 3. Mean rule acceptance was 4.136 (*SE* = 0.149) in the vote condition and 3.600 (*SE* = 0.172) in the no-vote condition. The Mann–Whitney U test indicated that this difference was statistically significant (*p* = 0.037). Table A9 reports the full-sample regression estimates. In the OLS models, the coefficient of vote condition was positive and statistically significant both without additional controls (*b* = 0.536, *SE* = 0.227, standardized *β* = 0.162, *p* = 0.019, 95% CI [0.087, 0.984]) and with controls for individual characteristics (*b* = 0.597, *SE* = 0.215, standardized *β* = 0.181, *p* = 0.006, 95% CI [0.173, 1.021]). The corresponding ordered logit estimates were likewise positive and statistically significant without additional controls (*b* = 0.522, *SE* = 0.249, *p* = 0.036, 95% CI [0.034, 1.011]) and with controls for individual characteristics (*b* = 0.692, *SE* = 0.251, *p* = 0.006, 95% CI [0.200, 1.185]). Thus, the positive effect of voting on rule acceptance remains when participants who failed the comprehension check are included.

**Table A9:** Effect of voting on rule acceptance in full sample.

	(1) Rule Acceptance OLS	(2) Rule Acceptance OLS	(3) Rule Acceptance Ordered Logit	(4) Rule Acceptance Ordered Logit
Vote condition	0.536 ** (0.227)	0.597 *** (0.215)	0.522 ** (0.249)	0.692 *** (0.251)
Individual characteristics	No	Yes	No	Yes
Constant	3.600 *** (0.171)	−0.048 (1.066)	—	—
*N*	208	208	208	208
*R*^2^/Pseudo *R*^2^	0.026	0.137	0.006	0.047

Note. The sample includes all 208 participants who completed Experiment 3, including the 68 participants who failed the comprehension check. The vote condition is coded as 1, with the no-vote condition serving as the reference category. Robust standard errors are reported in parentheses. Individual-characteristic controls include risk preference, gender, age, family location, and household per capita monthly income. ** *p* < 0.05; *** *p* < 0.01.

We also re-estimated the interaction models using all 208 initially recruited participants. Table A10 presents the results. For contribution, the interaction between the vote condition and the egalitarian index was negative and marginally significant in the OLS model without additional controls (*b* = −10.517, *SE* = 6.323, standardized *β* = −0.159, *p* = 0.098, 95% CI [−22.983, 1.949]). After controlling for individual characteristics, the interaction remained negative but fell just short of the 10% significance threshold (*b* = −10.339, *SE* = 6.303, standardized *β* = −0.157, *p* = 0.103, 95% CI [−22.769, 2.091]). The corresponding Tobit interaction estimates were negative and statistically significant both without additional controls (*b* = −17.546, *SE* = 8.474, *p* = 0.038, 95% CI [−34.155, −0.937]) and with controls for individual characteristics (*b* = −17.864, *SE* = 8.426, *p* = 0.034, 95% CI [−34.378, −1.349]). For rule acceptance, the interaction term was negative but not statistically significant in the unadjusted OLS model (*b* = −1.659, *SE* = 1.556, standardized *β* = −0.094, *p* = 0.288, 95% CI [−4.726, 1.409]) and was negative and marginally significant after controlling for individual characteristics (*b* = −2.241, *SE* = 1.333, standardized *β* = −0.126, *p* = 0.094, 95% CI [−4.869, 0.388]). The ordered logit interaction estimates were also negative but not statistically significant either without additional controls (*b* = −1.702, *SE* = 1.779, *p* = 0.339, 95% CI [−5.189, 1.785]) or with controls for individual characteristics (*b* = −2.639, *SE* = 1.697, *p* = 0.120, 95% CI [−5.965, 0.687]). Overall, the direction of the interaction remained consistent with the primary results, although the full-sample evidence was stronger for contribution than for rule acceptance.

**Table A10:** Interaction between voting and the pre-treatment egalitarian index in full Experiment 3 sample.

	(1) Contribution	(2) Contribution	(3) Rule Acceptance	(4) Rule Acceptance
Panel A. OLS				
Vote condition	1.999 ** (0.847)	1.916 ** (0.825)	0.524 ** (0.227)	0.600 *** (0.213)
Egalitarian index (centered)	1.838 (4.918)	2.502 (4.706)	0.080 (1.358)	0.753 (1.158)
Vote condition × egalitarian index	−10.517 * (6.323)	−10.339 (6.303)	−1.659 (1.556)	−2.241 * (1.333)
Individual characteristics	No	Yes	No	Yes
Constant	10.893 *** (0.647)	−2.470 (3.476)	3.599 *** (0.172)	−0.273 (1.098)
*N*	208	208	208	208
*R* ^2^	0.046	0.145	0.034	0.146
Panel B. Tobit and ordered logit				
Vote condition	3.129 ** (1.255)	3.036 ** (1.199)	0.524 ** (0.249)	0.710 *** (0.250)
Egalitarian index (centered)	3.589 (6.788)	4.724 (6.503)	0.105 (1.600)	1.215 (1.590)
Vote condition × egalitarian index	−17.546 ** (8.474)	−17.864 ** (8.426)	−1.702 (1.779)	−2.639 (1.697)
Demographic controls	No	Yes	No	Yes
Constant	11.375 *** (0.926)	−8.854 (5.522)	—	—
*N*	208	208	208	208
Pseudo *R*^2^	0.010	0.030	0.008	0.050

Note. The sample includes all 208 participants who completed Experiment 3, including the 68 participants who failed the comprehension check. The vote condition is coded as 1, with the no-vote condition serving as the reference category. Robust standard errors are reported in parentheses. Individual-characteristic controls include risk preference, gender, age, family location, and household per capita monthly income. * *p* < 0.10; ** *p* < 0.05; *** *p* < 0.01.

#### Appendix B.3.2. Controlling for Expected Peer Contribution

As a supplementary robustness check, Table A11 reports models that additionally control for expected peer contribution. Columns (1) and (2) present the results for contribution using OLS and two-limit Tobit models, respectively. The estimated effect of voting remained positive and was marginally significant at the 10% level in the OLS model (*b* = 1.764, *SE* = 1.058, standardized *β* = 0.137, *p* = 0.098, 95% CI [−0.329, 3.858]) and statistically significant in the two-limit Tobit model (*b* = 3.077, *SE* = 1.530, *p* = 0.044, 95% CI [0.079, 6.075]). Columns (3) and (4) report the corresponding OLS and ordered logit results for rule acceptance. The vote-condition estimate was also positive and marginally significant at the 10% level in both OLS (*b* = 0.513, *SE* = 0.283, standardized *β* = 0.149, *p* = 0.072, 95% CI [−0.047, 1.074]) and ordered logit models (*b* = 0.531, *SE* = 0.303, *p* = 0.079, 95% CI [−0.062, 1.125]). Overall, the positive effect of voting on contribution remained directionally consistent after controlling for expected peer contribution, although the statistical evidence was weaker.

**Table A11:** Effect of voting on contributions and rule acceptance after controlling for expected peer contribution.

	(1) Contribution OLS	(2) Contribution Tobit	(3) Rule Acceptance OLS	(4) Rule Acceptance Ordered Logit
Vote condition	1.764 * (1.058)	3.077 ** (1.530)	0.513 * (0.283)	0.531 * (0.303)
Individual characteristics	Yes	Yes	Yes	Yes
Expected peer contribution	Yes	Yes	Yes	Yes
Constant	−2.538 (4.254)	−9.690 (6.712)	0.090 (1.326)	—
*N*	140	140	140	140
*R*^2^/Pseudo *R*^2^	0.182	0.038	0.120	0.041

Note. All models control for expected peer contribution, risk preference, gender, age, family location, and household income category. The vote condition is coded as 1, with the no-vote condition serving as the reference category. Robust standard errors are reported in parentheses. * *p* < 0.10; ** *p* < 0.05.

We further re-estimated the interaction models after adding expected peer contribution together with controls for individual characteristics. As shown in Table A12, the interaction between the vote condition and the egalitarian index remained negative and marginally significant in the OLS contribution model (*b* = −14.532, *SE* = 8.485, standardized *β* = −0.187, *p* = 0.089, 95% CI [−31.319, 2.255]) and was negative and statistically significant in the corresponding Tobit model (*b* = −23.904, *SE* = 10.606, *p* = 0.024, 95% CI [−44.692, −3.116]). For rule acceptance, the interaction remained negative and statistically significant in both OLS (*b* = −4.467, *SE* = 1.740, standardized *β* = −0.214, *p* = 0.011, 95% CI [−7.910, −1.025]) and ordered logit models (*b* = −4.717, *SE* = 2.041, *p* = 0.021, 95% CI [−8.717, −0.717]). These results indicate that the interaction pattern remains after additionally controlling for expected peer contribution.

**Table A12:** Interaction between voting and pre-treatment egalitarian index after controlling for expected peer contribution.

	(1) Contribution OLS	(2) Contribution Tobit	(3) Rule Acceptance OLS	(4) Rule Acceptance Ordered Logit
Vote condition	1.920 * (1.063)	3.214 ** (1.503)	0.560 ** (0.275)	0.603 ** (0.301)
Egalitarian index (centered)	9.519 *** (3.105)	15.069 ** (6.217)	2.879 ** (1.253)	3.492 ** (1.584)
Vote condition × egalitarian index	−14.532 * (8.485)	−23.904 ** (10.606)	−4.467 ** (1.740)	−4.717 ** (2.041)
Individual characteristics	Yes	Yes	Yes	Yes
Expected peer contribution	Yes	Yes	Yes	Yes
Constant	−2.797 (4.245)	−10.521 (6.643)	0.007 (1.357)	—
*N*	140	140	140	140
*R*^2^/Pseudo *R*^2^	0.206	0.043	0.151	0.051

Note. All models control for expected peer contribution, risk preference, gender, age, family location, and household income category. The vote condition is coded as 1, with the no-vote condition serving as the reference category. Robust standard errors are reported in parentheses. * *p* < 0.10; ** *p* < 0.05; *** *p* < 0.01.

## Appendix C. Supplementary Evidence from a Nonbinding-Voice Condition

### Appendix C.1. Design and Procedure

To assess whether nonbinding voice alone could account for the effects observed in the vote condition, we added a follow-up nonbinding-voice condition. This condition used the same public goods task and outcome measures as the other conditions in Experiment 3 and differed only in the procedure preceding the public goods game. Before proceeding to the game, participants received the following instruction: “Please indicate whether you wish to take part in this interaction. You and the other three group members will each select either ‘participate’ or ‘do not participate.’ Please note that this opinion poll is used only to record your opinion and will not determine the subsequent arrangement.” All participants therefore proceeded to the public goods game, irrespective of the opinions expressed. This condition provided nonbinding voice without giving the expressed preferences a consequential role in determining participation.

We initially recruited 59 participants for this condition. A total of 11 participants failed at least one comprehension question, leaving 48 participants in the analytical sample. The final sample included 25 women and 23 men and had a mean age of 21.792 years (*SD* = 2.790). 34 participants (70.8%) reported an urban family location, and the median household per capita monthly income category was CNY 4001–6000.

### Appendix C.2. Analysis

We compared the 48 participants in the nonbinding-voice condition with the 58 participants in the original no-vote condition and the 82 participants in the original vote condition. Mean contribution was 10.345 tokens (*SE* = 0.774) in the no-vote condition, 10.458 tokens (*SE* = 0.854) in the nonbinding-voice condition, and 13.061 tokens (*SE* = 0.716) in the vote condition. The Mann–Whitney U test showed no difference between the nonbinding-voice and no-vote conditions (*p* = 0.574). Contributions were higher in the vote condition than in both the no-vote condition (*p* = 0.010) and the nonbinding-voice condition (*p* = 0.048).

We next estimated regression models with indicators for the nonbinding-voice and vote conditions, using the no-vote condition as the reference category. Columns (1) and (2) of Table A13 report the OLS estimates. Without additional controls, contribution did not differ significantly between the nonbinding-voice and no-vote conditions (*b* = 0.114, *SE* = 1.151, standardized *β* = 0.008, *p* = 0.922, 95% CI [−2.157, 2.384]). By contrast, contribution was higher in the vote condition than in the nonbinding-voice condition (*b* = 2.603, *SE* = 1.114, standardized *β* = 0.206, *p* = 0.021, 95% CI [0.406, 4.800]). After controls for individual characteristics were included, the nonbinding-voice condition still did not differ significantly from the no-vote condition (*b* = −0.561, *SE* = 1.239, standardized *β* = −0.039, *p* = 0.652), whereas contributions in the vote condition remained higher than those in the no-vote condition (*b* = 2.193, *SE* = 1.062, standardized *β* = 0.174, *p* = 0.040). The Tobit estimates in Columns (3) and (4) produced the same pattern. These exploratory results suggest that nonbinding voice alone is unlikely to explain the higher contributions observed in the vote condition.

**Table A13:** Effects of nonbinding voice and binding voting on contributions.

	(1) Contribution OLS	(2) Contribution OLS	(3) Contribution Tobit	(4) Contribution Tobit
Nonbinding-voice condition	0.114 (1.151)	−0.561 (1.239)	0.248 (1.639)	−0.798 (1.755)
Vote condition	2.716 ** (1.055)	2.193 ** (1.062)	4.349 *** (1.590)	3.592 ** (1.551)
Individual characteristics	No	Yes	No	Yes
Constant	10.345 *** (0.774)	0.082 (3.670)	10.522 *** (1.106)	−4.461 (5.516)
*N*	188	188	188	188
*R*^2^/Pseudo *R*^2^	0.045	0.103	0.009	0.021

Note. Nonbinding-voice condition and Vote condition are indicator variables for the corresponding groups; the no-vote condition is the reference group (both indicators equal 0). Robust standard errors are reported in parentheses. Individual-characteristic controls include risk preference, gender, age, family location, and household per capita monthly income. ** *p* < 0.05; *** *p* < 0.01.

Mean rule acceptance was 3.534 (*SE* = 0.212) in the no-vote condition, 3.583 (*SE* = 0.222) in the nonbinding-voice condition, and 4.146 (*SE* = 0.192) in the vote condition. Mann–Whitney U tests indicated that rule acceptance was marginally lower in the no-vote condition than in the vote condition (*p* = 0.067), whereas there was no significant difference between the no-vote and nonbinding-voice conditions (*p* = 0.831). The difference between the nonbinding-voice and vote conditions was also not statistically significant (*p* = 0.126).

Table A14 reports regression estimates for rule acceptance, using the no-vote condition as the reference category. In OLS specifications, the coefficient of nonbinding-voice condition was not statistically significant either without additional controls (*b* = 0.049, *SE* = 0.307, standardized *β* = 0.013, *p* = 0.874, 95% CI [−0.556, 0.654]) or with controls for individual characteristics (*b* = −0.076, *SE* = 0.317, standardized *β* = −0.020, *p* = 0.810). The coefficient of vote condition was positive and statistically significant without controls (*b* = 0.612, *SE* = 0.286, standardized *β* = 0.182, *p* = 0.034, 95% CI [0.048, 1.176]) and with controls for individual characteristics (*b* = 0.561, *SE* = 0.281, standardized *β* = 0.167, *p* = 0.047). In the ordered logit models, the coefficient of vote condition was positive and marginally significant both without controls (*b* = 0.567, *SE* = 0.308, *p* = 0.066) and with controls (*b* = 0.547, *SE* = 0.297, *p* = 0.065), whereas the coefficient of nonbinding-voice condition was not statistically significant in either specification (*ps* > 0.8). These exploratory results suggest that nonbinding voice alone may not fully explain the higher rule acceptance observed in the vote condition.

**Table A14:** Effects of nonbinding voice and binding voting on rule acceptance.

	(1) Rule Acceptance OLS	(2) Rule Acceptance OLS	(3) Rule Acceptance Ordered Logit	(4) Rule Acceptance Ordered Logit
Nonbinding-voice condition	0.049 (0.307)	−0.076 (0.317)	0.076 (0.339)	−0.061 (0.352)
Vote condition	0.612 ** (0.286)	0.561 ** (0.281)	0.567 * (0.308)	0.547 * (0.297)
Individual characteristics	No	Yes	No	Yes
Constant	3.534 *** (0.212)	1.557 (1.132)	—	—
*N*	188	188	188	188
*R*^2^/Pseudo *R*^2^	0.031	0.074	0.006	0.022

Note. Nonbinding-voice condition and Vote condition are indicator variables for the corresponding groups; the no-vote condition is the reference group (both indicators equal 0). Robust standard errors are reported in parentheses. Individual-characteristic controls include risk preference, gender, age, family location, and household per capita monthly income. * *p* < 0.10; ** *p* < 0.05; *** *p* < 0.01.

We repeated the analyses using all 267 initially recruited participants, including those who failed the comprehension check. The substantive pattern was unchanged. Contribution in the nonbinding-voice condition did not differ from that in the no-vote condition, whereas the vote condition had higher contributions than both comparison conditions. Taken together, the exploratory pilot results are inconsistent with interpreting the contribution difference as a consequence of nonbinding voice alone.
